# Derailed degradation: LRRK2-dependent exocytosis in Parkinson’s disease

**DOI:** 10.1073/pnas.2515758122

**Published:** 2025-08-04

**Authors:** Shawn M. Ferguson

**Affiliations:** ^a^Department of Cell Biology, Yale University School of Medicine, New Haven, CT 06510; ^b^Department of Neuroscience, Program in Cellular Neuroscience, Neurodegeneration and Repair, Wu Tsai Institute, Kavli Institute for Neuroscience, Yale University School of Medicine, New Haven, CT 06510; ^c^Aligning Science Across Parkinson’s Collaborative Research Network, Chevy Chase, MD 20815

It is well established that increased kinase activity of leucine-rich repeat kinase 2 (LRRK2) confers Parkinson’s disease risk (1–4). The robust links between LRRK2 and Parkinson’s disease have motivated the development of candidate therapeutic strategies designed to limit LRRK2 kinase activity and have raised important questions about the functions of LRRK2 and how they contribute to disease-causing cellular processes (5–7). In PNAS, Palumbos et al. present new data showing impacts of Parkinson’s disease–associated gain-of-function LRRK2 mutations on fates of cargos that have been captured within neuronal endosomes and autophagosomes (8). Analysis of the media from cultures of mouse and human cortical neurons with disease-causing LRRK2 mutations revealed an increased abundance of extracellular vesicles. Characterization of their sizes, as well as biochemical, proteomic, and transcriptomic analyses of their contents, provided insights into the origins of these extracellular vesicles.

Small vesicles enriched in intralumenal endosomal components were more abundant in media from LRRK2 mutant neurons, reflecting increased exocytosis of multivesicular bodies (Fig. 1). Meanwhile, the larger secreted vesicles had a composition that overlapped with recently defined internal contents of neuronal autophagosomes and thus reflected their LRRK2-dependent exocytosis (9). These discoveries that link disease-causing LRRK2 mutations to striking neuronal exocytosis phenotypes provide insights into the cellular impacts of LRRK2 dysregulation and also highlight critical gaps in our understanding. The subsequent paragraphs will address relationships between endosomes, autophagosomes, and lysosomes, candidate sites of action of LRRK2 and potential physiological and pathophysiological impacts of LRRK2-dependent control of lysosomal trafficking and quality control.

**Fig. 1.**
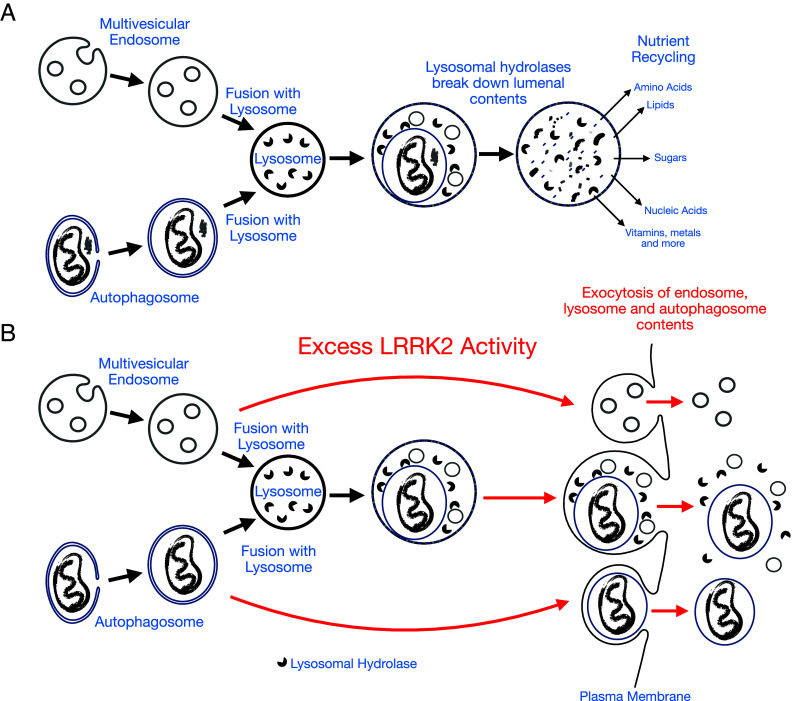
Intersection between excessive LRRK2 activity and exocytosis of endosome, autophagosome, and lysosome contents. (*A*) The endocytic and autophagic pathways have unique mechanisms for capturing cargos which they deliver to lysosomes for degradation. This process coordinates the clearance of potentially toxic damaged organelles, protein aggregates, and transmembrane proteins with the recycling of nutrients. (*B*) In cells with elevated LRRK2 activity, increased exocytosis of multivesicular bodies, autophagosomes, and/or lysosomes results in the release of their contents to the extracellular space. The precise mechanisms that mediate these LRRK2-dependent effects and the relative amounts of exocytosis of each organelle remain to be established across specialized cell types.

Although late endosomes and autophagosomes differ in their origins and in the cargos that they contain, they normally share a common fate, fusion with lysosomes to enable macromolecule degradation and nutrient recycling (Fig. 1*A*). Palumbos et al. propose that endosomes and autophagosomes independently undergo exocytosis in response to impaired lysosome functions arising from Parkinson’s disease–associated mutations in LRRK2 (8). In this model, a defect in the ability of endosomes and autophagosomes to fuse with dysfunctional lysosomes leads them to instead fuse with the plasma membrane (Fig. 1*B*). This could help to reduce the accumulation of waste in a cell that is struggling to keep up with demands on its lysosomes. However, it is also possible that LRRK2 plays a more direct role in promoting the exocytosis of lysosomes. For example, exocytosis of endosomal and autophagosomal cargos could occur following their fusion with lysosomes and would be accompanied by the release of lysosomal hydrolases (Fig. 1*B*). In this scenario, LRRK2-dependent defects in lysosome degradative activity along with promotion of lysosome exocytosis would explain the increased abundance of membrane-enclosed cargos derived from both the endocytic and autophagic pathways.

Increased exocytosis of cargos destined for lysosomes from neurons with gain-of-function LRRK2 mutations parallels responses of other cell types to LRRK2 perturbations. LRRK2 knockout mice accumulate abnormal lysosomes in kidney proximal tubules and in humans the urinary abundance of lipids associated with exocytosis of endolysosomal intraluminal vesicles correlated with LRRK2 kinase activity (10, 11). In lungs, LRRK2 is highly expressed in type 2 pneumocytes and studies ranging from knockout rodents to primates support a role for LRRK2 in the biology of lamellar bodies, a lysosome-related organelle whose exocytosis mediates the secretion of surfactant (10). LRRK2 also promotes lysosome exocytosis and release of lysosomal hydrolases in macrophages following treatment with drugs that perturb lysosome function (12). Thus, a relationship between LRRK2 and exocytosis of lysosomes and lysosome-related organelles is generalizable across multiple cell types.

Recent studies have revealed mechanistic insights into how LRRK2 physically interacts with lysosomes. The lysosome stress response known as conjugation of ATG8 to single membranes (CASM) activates LRRK2 on the surface of distressed lysosomes via a direct interaction between LRRK2 and the ATG8 family member known as GABARAP (7, 13, 14). This pathway is critical for lysosome exocytosis in response to lysosome damage in macrophages (14). However, it remains to be determined to what extent this CASM-dependent LRRK2–GABARAP interaction supports LRRK2-driven exocytosis in neurons. LRRK2 activation at organelle membranes is also promoted by interactions with multiple Rab guanosine triphosphatases (GTPases) (4). These LRRK2–Rab interactions may additionally contribute to LRRK2 activity at the surface of endolysosomal organelles prior to their exocytosis (12).

A major breakthrough in understanding cellular functions of LRRK2 was the identification of specific Rab GTPases (Rabs 3A,B,C,D, 8A/B, 10, 12, 35, and 43) as LRRK2 substrates (4). These Rab proteins represent strong candidates for mediating downstream functions of LRRK2. Importantly, several of the Rabs that are phosphorylated by LRRK2 (including Rabs 3, 8, and10) play roles in exocytosis (15). However, the specific contributions of LRRK2 phosphorylation of these Rabs to exocytosis of endolysosomes and autolysosomes have not been systematically defined across different cell types, and their contributions to this process in neurons require further investigation.

In addition to promoting exocytosis, excessive neuronal LRRK2-dependent Rab phosphorylation was previously linked to impaired coordination of processes that control retrograde axonal transport and maturation of autophagosomes into lysosomes (16, 17). It thus remains unclear whether LRRK2 directly promotes exocytosis of endolysosomal organelles in neurons by phosphorylating Rabs that engage the exocytic machinery, or whether increased exocytosis reflects a compensatory response to impaired lysosomal degradation, allowing cells to offload excess cargo when lysosomes are overwhelmed.

Given the extreme polarity of neurons, LRRK2 may play distinct roles in axons, dendrites, and neuronal cell bodies. It was noted that exocytosis of MVBs occurred more prominently in neuronal cell bodies versus autophagosome-related exocytosis that showed an increased likelihood to take place in proximal dendrites (8). However, it is unknown whether this reflects different subcellular functions and/or regulation of LRRK2 versus more general properties of these organelles. These questions emphasize the importance of further fundamental cell biological research into how quality control of the autophagic and endolysosomal pathways is compartmentalized in neurons.

Although much remains to be discovered concerning LRRK2 and the role of exocytosis in protecting neurons from excess load on their lysosomes, it is possible to speculate about in vivo physiological and pathophysiological relevance of these processes. Exocytic release of the contents of endosomes and autophagosomes may relieve stress arising from impaired macromolecular clearance when excessive LRRK2 kinase activity impairs the degradative activity of lysosomes (18). Along these lines, neuronal disposal of protein aggregates and mitochondria via shedding from neurons for clearance by neighboring glial cells has been observed in other contexts but has not yet been linked to LRRK2 or its aberrant activity in Parkinson’s disease (19).

In PNAS, Palumbos et al. present new data showing impacts of Parkinson’s disease-associated gain-of-function LRRK2 mutations on fates of cargos that have been captured within neuronal endosomes and autophagosomes.

Of potentially high relevance in the context of Parkinson’s disease, the cell-to-cell spread of pathogenic α-synuclein fibrils is thought to be a central factor in disease pathology that has multiple points of connection with autophagy and the endolysosomal pathway (20). Autophagic capture of cytoplasmic α-synuclein fibrils followed by fusion with lysosomes and exocytic release of the α-synuclein prior to its degradation by lysosomal proteases provides an extracellular escape route for α-synuclein. These intimate connections between α-synuclein, autophagy, and the endolysosomal pathway point to places where LRRK2-driven changes in lysosomal degradative activity and exocytosis have the potential to promote the pathogenic spread of α-synuclein fibrils.

In summary, recent years have yielded important insights that have sharpened focus on LRRK2 functions that affect the ability of lysosomes to degrade macromolecules and recycle nutrients. This supports future studies to define physiological and pathophysiological consequences of excessive LRRK2 activity arising from lysosome damage and Parkinson’s disease–related mutations. Progress on these topics is critical for both understanding the cellular basis for Parkinson’s disease and for designing and testing new therapies that target LRRK2 and its associated biology.

## Data Availability

No new primary data were collected in this study. No code was generated for this study.
